# Structure of the substrate-engaged SecA-SecY protein translocation machine

**DOI:** 10.1038/s41467-019-10918-2

**Published:** 2019-06-28

**Authors:** Chengying Ma, Xiaofei Wu, Dongjie Sun, Eunyong Park, Marco A. Catipovic, Tom A. Rapoport, Ning Gao, Long Li

**Affiliations:** 1 State Key Laboratory of Membrane Biology, Peking-Tsinghua Center for Life Sciences, School of Life Sciences, Peking University, Beijing, China; 2 University of California-Berkeley, Stanley Hall, Berkeley, CA 94720 USA; 3 Department of Cell Biology, Howard Hughes Medical Institute and Harvard Medical School, 240 Longwood Avenue, Boston, MA 02115 USA

**Keywords:** Protein translocation, Cryoelectron microscopy

## Abstract

The Sec61/SecY channel allows the translocation of many proteins across the eukaryotic endoplasmic reticulum membrane or the prokaryotic plasma membrane. In bacteria, most secretory proteins are transported post-translationally through the SecY channel by the SecA ATPase. How a polypeptide is moved through the SecA-SecY complex is poorly understood, as structural information is lacking. Here, we report an electron cryo-microscopy (cryo-EM) structure of a translocating SecA-SecY complex in a lipid environment. The translocating polypeptide chain can be traced through both SecA and SecY. In the captured transition state of ATP hydrolysis, SecA’s two-helix finger is close to the polypeptide, while SecA’s clamp interacts with the polypeptide in a sequence-independent manner by inducing a short β-strand. Taking into account previous biochemical and biophysical data, our structure is consistent with a model in which the two-helix finger and clamp cooperate during the ATPase cycle to move a polypeptide through the channel.

## Abstract (web summary)

Proteins are translocated across membranes through the Sec61/SecY channel. Here, the authors present the structure of a translocating peptide chain trapped inside the SecA-SecY complex which suggests how peptides are actively moved through the channel.

## Introduction

Protein translocation is a universal and essential process that allows the export of secretory proteins from cells and the integration of membrane proteins into lipid bilayers^1–6^. The central translocation component is an evolutionarily conserved protein-conducting channel, the Sec61 channel in eukaryotes and the SecY channel in prokaryotes. Both are formed from a heterotrimeric protein complex, containing a large subunit with ten trans-membrane (TM) helices (Sec61α in eukaryotes and SecY in prokaryotes), and two small subunits that in most species contain only one TM segment (called Sec61β and Sec61γ in eukaryotes and SecG and SecE in bacteria). The Sec61β/SecG subunit is not essential for the function of the channel^7^. The Sec61/SecY channel has an hourglass shape, with an empty cytosolic funnel and an extracellular funnel that is filled with a plug domain^8–11^. A constriction is formed in the middle of the channel by a ring of conserved hydrophobic amino acids, called the pore ring. During translocation, the plug is displaced, and the polypeptide chain moves through the pore ring across the membrane. A lateral gate in Sec61α/SecY allows hydrophobic signal sequences of secretory proteins or TM segments of membrane proteins to exit into the lipid phase.

The channel needs to associate with a partner that provides the driving force for translocation (for review, see ref. ^5^). In co-translational translocation, the Sec61/SecY channel associates with the translating ribosome, such that a nascent polypeptide is moved from the ribosome tunnel into the membrane channel. In posttranslational translocation in eukaryotes, the Sec61 channel partners with another membrane protein complex, the Sec62/63 complex, as well as with the luminal chaperone BiP, a member of the Hsp70 family of ATPases. BiP binds to the polypeptide as it emerges into the ER lumen and prevents it from sliding back into the cytosol^12^. In posttranslational translocation in bacteria, the SecY channel associates with the SecA ATPase. SecA uses the energy of ATP hydrolysis to move polypeptides through the channel, but the mechanism of translocation is poorly understood.

SecA has two nucleotide-binding domains (NBD1 and NBD2), which bind the nucleotide at their interface^13^. A two-helix finger (THF), consisting of two helices connected by a loop, inserts into the cytoplasmic funnel of the SecY channel^14,15^. A clamp, formed by rotation of the polypeptide-cross-linking domain (PPXD) toward NBD2, positions the translocating polypeptide chain above the channel^16^.

Several mechanisms have been proposed for SecA. In a ratcheting model^17,18^, SecA’s THF senses bulky amino acid residues of a substrate. When it encounters such a residue, SecA converts from the ADP-bound to the ATP-bound state and the SecY channel opens, allowing the residue to move through the pore. Following ATP hydrolysis, the channel closes, trapping the bulky residue on the other side of the pore ring. In a power-stroke model^19^, ATP binding of SecA causes the THF to move toward the channel and push the polypeptide chain into the channel; following ATP hydrolysis, the finger retracts and allows sliding of the chain in either direction. During retraction of the THF, the clamp holds the polypeptide chain, so that the chain is not dragged backward when the THF resets^14,20^. In the originally proposed version of a power-stroke model, large domains of SecA reach entirely through membrane to deliver the substrate to the other side^21,22^.

An understanding of the mechanism of posttranslational protein translocation requires structures of the active Sec61/SecY channel and visualization of the translocating polypeptide substrate. Cross-linking experiments provided evidence that a substrate contacts both the clamp and the THF^16^, but the exact path of the translocating chain could not be deduced. A previously determined crystal structure contained the detergent-solubilized SecY channel, the SecA ATPase, and a segment of a secretory protein (proOmpA) fused into the THF of SecA (SecA-OAins)^23^. The substrate segment inserted as a loop into the channel, with the signal sequence forming a helix outside the lateral gate. However, the construct design did not allow determination of the polypeptide path through SecA, and it is even unclear to what extent the artificial nature of the construct affected the position of the substrate in SecY. Here, we report a structure of an active translocon with a translocating polypeptide chain caught in the act of moving through SecA and SecY.

## Results

### Assembly of an active translocation complex

**Fig. 1:**
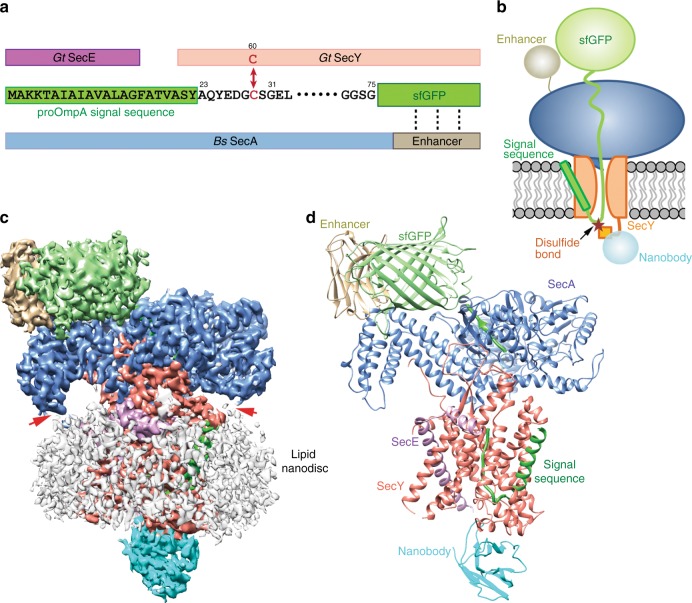
Overall structure of the SecA-SecY-substrate complex **a** Components of the translocation complex. The protein sequences are shown schematically as bars in different colors. *G. thermodenitrificans* (Gt) SecE, purple; SecY, salmon; sfGFP, green; *B. subtilis* (Bs) SecA, blue; and Enhancer, tan. The proOmpA signal sequence is highlighted in green. Cysteines (C) used for cross-linking (double arrow) are shown in red. The interaction between sfGFP and Enhancer are indicated by three dashed lines. The positions of some residues are indicated. **b** Scheme of the assembled translocation complex. **c** Cryo-EM density map of the SecA-SecY-substrate complex, with density for the individual proteins colored as in (**a**) and (**b**). The lipid nanodisc is colored in gray. Interaction sites between SecA and the lipid surface are indicated by red arrowheads. **d** Ribbon model of the complex. Individual proteins are colored and labeled as in (**a**) and (**b**)

To obtain a structure of a translocating SecA-SecY-substrate complex, we assembled in *E. coli* cells a complex containing *Bacillus subtilis* SecA (residues 1–778), *Geobacillus thermodenitrificans* SecYE, and a translocating polypeptide substrate. SecA lacks C-terminal residues, which are dispensable for its function^24^. The substrate consists of the signal sequence of proOmpA, a linker polypeptide segment, and superfolder Green Fluorescence Protein (sfGFP)^25^ (Fig. 1a, b). The bulky sfGFP moiety prevents the C-terminus of the substrate from moving through the SecA-SecY complex, thus generating a translocation intermediate. To reduce the flexibility of the GFP moiety, an anti-GFP nanobody (enhancer)^26^ was fused to the C-terminus of SecA. Stabilization of the channel-inserted polypeptide was achieved by a disulfide bridge formed without addition of an exogenous oxidant; it linked a cysteine introduced C-terminally of the signal sequence to a cysteine placed into the plug domain of SecY (Fig. 1a, b). The complex was purified in the presence of ADP·BeFx to lock SecA in the transition state of ATP hydrolysis. The complex was then reconstituted into nanodiscs^27^, generating a physiological lipid environment for the channel (Supplementary Fig. 1). Before analysis by cryo-EM, an anti-SecY nanobody was added to reduce the flexibility of the channel on its periplasmic side^23^. Stabilization agents such as nanobodies and disulfide cross-links proved crucial to maintain the translocation complex for crystallographic studies, though it is unclear whether they improved the cryo-EM map quality. A cryo-EM density map was obtained with an overall resolution of 3.5 Å (Fig. 1c, Supplementary Fig. 2, and Supplementary Table 1). The obtained map was of sufficient quality to build atomic models for SecA and SecYE (Fig. 1d), including segments that were poorly resolved in previous structures^14,23^, such as the loop between TM6 and TM7 of SecY (Supplementary Fig. 3). Most of the polypeptide substrate could also be traced (Fig. 1d).

### SecA and SecY in the active translocation complex

**Fig. 2:**
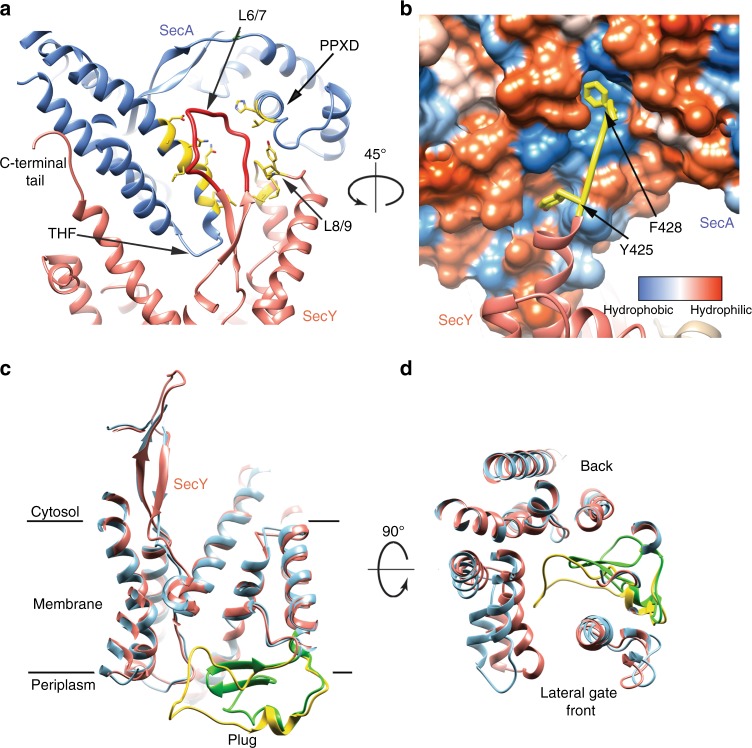
The interaction between SecA and SecY and changes in SecY’s plug domain. **a** Interaction of the loop between TMs 6 and 7 of SecY (L6/7) (red) with the PPXD (blue), the THF, and the loop between TMs 8 and 9 (L8/9). The regions involved are colored yellow. **b** Interaction of the C-terminal tail (yellow) of SecY with SecA. SecA is shown as a space-filling model with residues colored according to their hydrophobicity. Interacting SecY residues are shown as yellow sticks. **c** Comparison between the plug regions in the cryo-EM structure and the crystal structure of SecA-OAins/SecY (PDB ID: 5EUL). The SecY molecule and plug are colored salmon and yellow, respectively, for the cryo-EM structure, and cyan and green, respectively, for the crystal structure. **d** Top view of (**c**)

In the active translocation complex, the overall conformations of SecA and SecY are similar to those in previous structures^14,23^. SecA binds to several cytosolic loops of SecY, including the loop between TM8 and TM9 (L8/9), which interacts with the PPXD, and the C-terminal tail, which binds through the conserved hydrophobic residues Y425 and F428 into a groove in the helical scaffold domain (HSD) (Fig. 2a, b, Supplementary Fig. 3g). The loop between TM6 and TM7 of SecY (L6/7) has a previously unrecognized role in SecA interaction. It is surrounded by L8/9 of SecY, and by the THF and PPXD of SecA (Fig. 2a). Both SecY loops together help to induce a closed conformation of SecA’s clamp. SecA also interacts with the lipid surface of the nanodiscs. It binds through two charged regions (Fig. 1c), one including residues R553, R576, and K583 in the HSD, and the other including residues R645 and E646 in the helical wing domain (HWD) (Supplementary Fig. 4a and b). The N-terminal 13 amino acids of SecA, implicated for *E. coli* SecA in lipid binding^19,28^, are invisible and thus likely flexible.

The plug in the SecY channel occupies a more central position than in the SecA-OAins/SecY crystal structure and adopts a distorted helical conformation, rather than forming the previously observed β-strands (Fig. 2c, d). Although the cause for the conformational change is unclear, the plug can adopt different structures in different species and reforms from neighboring segments when deleted^29^. It thus appears that the only important function of the plug is to seal the channel in its closed state. The SecY channel is surrounded by lipids from the nanodisc (Fig. 1c). Some phospholipid molecules seem to be bound specifically, including one at the back of the channel (Supplementary Figs 5a and b), which is positioned parallel to the membrane surface with the hydrophilic head group deep inside the extracellular funnel. It might thus interact with the translocating polypeptide chain, although its functional significance remains unclear.

### The polypeptide substrate in the SecY channel

**Fig. 3:**
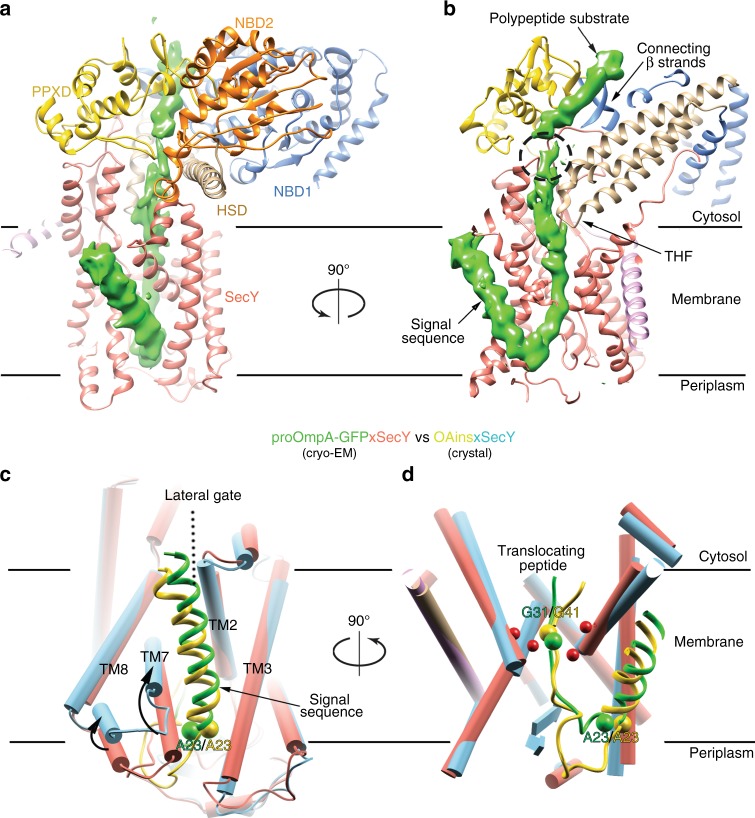
The translocating polypeptide in the SecA-SecY complex. **a** Density for the polypeptide (green) is shown in a ribbon diagram of the translocation complex. SecA domains are shown in different colors. **b** As in (**a**), but a cut-away view from a different angle. The dashed circle indicates a region where the substrate could not be traced, likely because it is flexible. **c** Comparison of the signal sequences and lateral gates between the cryo-EM and crystal (PDB ID: 5EUL) structures. SecY is shown as cylinders in salmon and cyan, respectively. The signal sequences are shown as ribbon diagrams, colored green and yellow for the cryo-EM and crystal structures, respectively. The movements of TM7 and TM8 are indicated by arrows. **d** As in (**c**), but cut-away side view from a different angle. The first residue after the signal sequence (Ala23) and the Gly residues trapped inside the pore rings (red balls) are labeled and shown as balls

The structure shows how the polypeptide substrate is translocated through the SecA-SecY complex (Fig. 3a, b). The N-terminal signal sequence forms a helix that is tilted by ~45° with respect to the membrane plane and bound to a groove on the outside of the lateral gate of the SecY channel, as in the SecA-OAins/SecY crystal structure^23^. However, the helix is shifted by half a turn toward the cytosolic side of the membrane (Fig. 3c). To accommodate this change, TMs 7 and 8 of SecY are tilted by ~10° toward the cytosolic side and the extracellular ends of TMs 7 and 3 approach each other, closing the lateral gate on the periplasmic side. These differences indicate that the signal sequence binding site might be flexible or was somewhat distorted in the previous artificial construct. Although bound to a groove of the SecY channel, the hydrophobic part of the signal sequence is exposed to the lipids of the nanodisc, supporting the idea that it is recognized mainly by partitioning into the lipid phase. This is consistent with the ability of signal sequences to be cross-linked to lipids^30,31^ and with the correlation between the partitioning of synthetic peptides into hydrophobic solvents and their function as signal sequences in vivo^32^. Lipid partitioning explains why signal sequences can vary in length and sequence, but require a hydrophobic peptide segment^23^. Some lipids seem to bind to certain sites at the interface to SecY (Supplementary Fig. 5c), perhaps participating in the interaction between the signal sequence and channel.

The polypeptide segment following the signal sequence makes a U-turn in the extracellular funnel. The loop is significantly shorter than in the crystal structure (9 versus 19 residues, counting from the first residue after the signal sequence, Ala23, to the residue in the pore ring (Fig. 3d). The shorter extracellular loop could correspond to an earlier stage of translocation, in which fewer residues of the C-terminal part of the loop have moved across the membrane. In both structures, a glycine residue is trapped in the pore ring (Gly31 and Gly41, in the present and previous structures, respectively). The pore ring residues of SecY form a gasket-like seal around these glycine residues (Supplementary Fig. 5d), preventing even small molecules, such as ions, to permeate through the membrane during translocation^33^. Glycines are probably trapped in the structures because they minimize pore expansion in the presence of a translocating polypeptide and thus lead to a more stable state. The polypeptide adopts an extended conformation inside the SecY channel. The polypeptide chain is almost perpendicular to the plane of the lipid bilayer and extends from the loop in the extracellular space to the cytoplasmic side of the membrane (Fig. 3b, d).

### The polypeptide substrate in SecA

The polypeptide chain could be traced all the way from SecY back to its entry point into SecA. The least resolved region is a small segment at the SecY-facing end of SecA’s clamp (encircled in Fig. 3b), where density for the polypeptide was only visible at lower thresholds, suggesting that this segment is flexible when it passes through. The connection between the polypeptide substrate and the C-terminal sfGFP is also flexible, although both the sfGFP moiety and the interacting SecA-fused nanobody are visible in the structure (Fig. 1c).

**Fig. 4:**
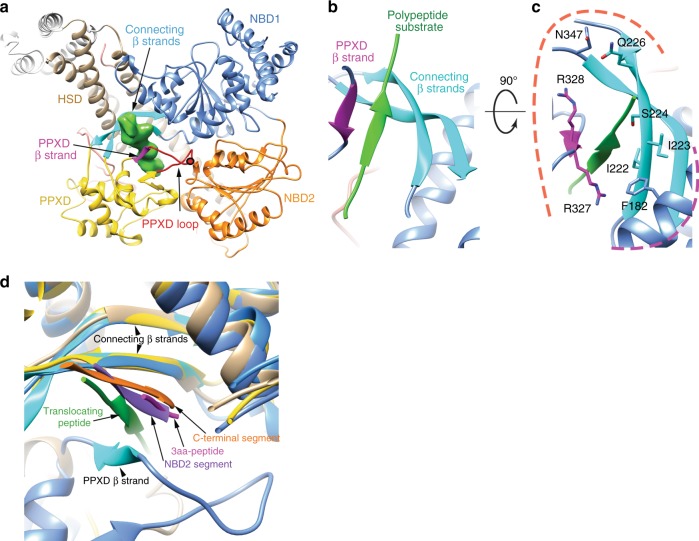
Polypeptide substrate in SecA. **a** Top view of the SecA-SecY-substrate complex, showing polypeptide entry into SecA. The SecY channel is underneath SecA and not shown. Substrate density is shown in green. The conserved Arg367 in the PPXD loop is indicated as a red dot. **b** Close-up view of the β-sheet formed by substrate binding. The connecting β-strands at the back of the clamp are in shown in cyan, the PPXD β-strand in magenta, and the β-strand formed by the substrate in green. **c** SecA residues surrounding the polypeptide substrate are shown as sticks. Hydrophilic and hydrophobic residues, clustered on different sides, are highlighted with orange and purple dashed curves. **d** Interaction of the connecting β strands with different peptides. Shown is a superposition of different structures, in which β-strands were induced. The alignment is based on the connecting β-strands. The peptides are derived from (1) the translocating peptide in the present structure (green); (2) a C-terminal SecA segment (orange, PDB 1M74); (3) three amino acids of a synthetic peptide (red, PDB 3JV2); and (4) a segment of NBD2 of a neighboring SecA molecule in the crystal (purple, PDB 2IBM)

At its entry point into SecA, the polypeptide chain is embraced by the clamp, formed from segments of the PPXD and HSD (Fig. 4a). Contrary to a previous prediction^14^, the NBD2 does not contact the polypeptide. On one side of the polypeptide path, a loop of the PPXD (residues 314–325) reaches into a cavity between NBD1 and NBD2 (Fig. 4a) and contains the highly conserved Arg367 residue (numbering for *B. subtilis* SecA; Fig. 4a), the mutation of which impairs translocation in *E. coli*^34^. On the other side of the path, in the back of the clamp, are two β-strands that connect NBD1 with the PPXD. Both the substrate and a segment right after the PPXD loop (residues 326–331) adopt short β-strands, so that the four strands together form a β-sheet (Fig. 4a, b, Supplementary Fig. 3i and j). The β-strand in PPXD is caused by the presence of substrate, as it is not seen in previous structures, including those of *E. coli* and *M. tuberculosis* SecA^13,35,36^. Similarly, the β-strand in the substrate itself is also induced. In fact, crystal structures of SecA in isolation showed that the normally unstructured C-terminus of SecA^13^ or an α-helix from a neighboring SecA molecule^37^ are induced to form β-strands that augment the two connecting β-strands. A small peptide also binds to the same site in a C-terminally truncated SecA construct^38^. The β-sheets in all these structures are superimposable although they contain different amino acid sequences (Fig. 4d), as expected for β-strands interacting by H-bonding of the polypeptide backbones.

**Fig. 5:**
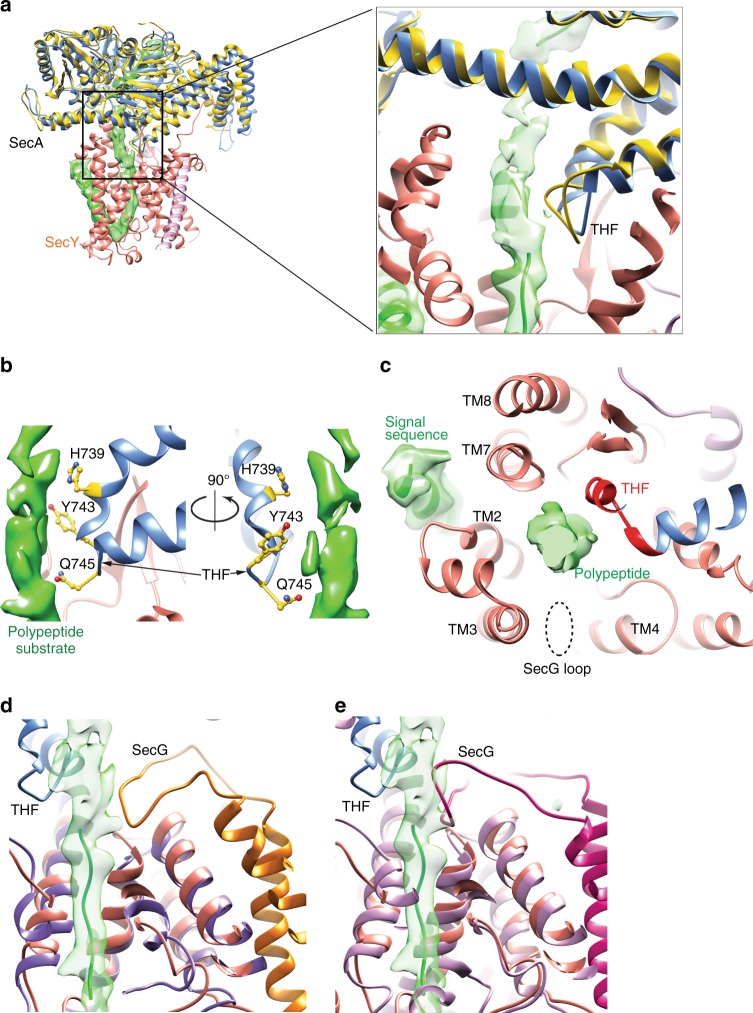
The polypeptide segment close to the THF. **a** Superposition of *B. subtilis* SecA (blue) in the current cryo-EM structure with *T. maritima* SecA (yellow) in the SecA-SecYEG crystal structure (PDB ID: 3DIN). SecYE and substrate density are from the current structure, while SecYEG and substrate from the crystal structure were deleted. The right panel shows a magnified view of the boxed area. **b** Close-up view of THF residues mediating substrate interaction (balls and sticks). **c** Top view of the polypeptide substrate close to the THF. Density for the substrate is shown in green and the surrounding TMs of SecY in ribbon representation. SecG, which is absent from the present structure, would be located at the position indicated by a dashed oval. **d** Superposition of *G. thermodenitrificans* SecYE in the current cryo-EM structure of the active channel with *T. maritima* SecYEG in a crystal structure of the SecA-primed channel (PDB ID: 3DIN). *G. thermodenitrificans* SecY is in salmon, *T. maritima* SecY is in purple, and *T. maritima* SecG is in gold. **e** Superposition of *G. thermodenitrificans* SecYE in the cryo-EM structure of the active channel with *T. thermophilus* SecYEG in a crystal structure of the idle channel (PDB ID:5AWW). *T. thermophilus* SecY is in purple and SecG is in magenta. The SecG loop clashes with the polypeptide density

The substrate segment closest to the THF shows some variability in its conformation, suggesting that it does not strongly interact with the THF; the polypeptide has a rather fragmented density in this region and it occupies slightly different positions in different 3D classes (Supplementary Fig. 6). The THF itself has a similar position as in the SecA-SecYEG crystal structure^14^ (Fig. 5a). Several residues of the THF (H739, Y743, and Q745) are in the vicinity of the polypeptide (Fig. 5b; Supplementary Fig. 3h), but they do not make strong contacts. Together with cytosolic loops of SecY, the THF guides the polypeptide chain into the channel (Figs. 3b, 5c). The SecG subunit of the SecY complex, omitted in our structure, may also play a role, as its cytosolic domain blocks the SecY channel in its idle state^10^ and would be close to the polypeptide substrate in the active channel (Fig. 5d, e).

## Discussion

Our cryo-EM structure shows the path of a translocating polypeptide chain from its entry point into the SecA ATPase all the way to the extracellular side of the SecY channel. The structure therefore approximates better a translocating state of the polypeptide chain than a previous crystal structure, in which a substrate segment was fused into the THF of SecA^20^. Nevertheless, it is possible that some distortion of the truly translocating state was caused in our structure by the use of nanobodies or disulfide bridge cross-linking. Cryo-EM structures have also been reported for active, ribosome-associated mammalian Sec61 channels, but the translocating polypeptide chains were invisible inside the channel^39–41^.

Our cryo-EM structure and previous crystal structures^13,37,38^ indicate that SecA induces the polypeptide substrate to form a short β-strand that augments a β-sheet at the back of the clamp. Binding is independent of the specific amino acid sequence, as it involves only the polypeptide backbone, a conclusion that is supported by the fact that several different peptides are induced to form β-strands (Fig. 4d). This interaction would allow any substrate segment entering SecA to bind, which may contribute to the ability of SecA to translocate a broad range of diverse polypeptides. However, some amino acid side chains of SecA’s clamp seem to interact with the polypeptide chain (e.g., R327, F182, and S224; Fig. 4c), which suggests that there may be some preference of the clamp for certain substrate sequences^19,42–44^.

Our structure cannot discriminate between the power-stroke and ratcheting models for SecA function. However, recent single-molecule FRET experiments support a power-stroke model^20^, as the THF is closest to the SecY channel when SecA is in the ATP-bound state, at an intermediate position in the transition state of ATP hydrolysis (ADP·BeFx), and farthest away in the ADP-bound state. These data imply that the actual power-stroke occurs during ATP binding to SecA, when the THF tip interacts with the polypeptide and pushes it into the SecY channel.

In our structure, SecA is in the transition state of ATP hydrolysis (ADP·BeFx). Consistent with the single-molecule FRET data, which indicate that in this nucleotide state the THF should be retracting from the channel, we observe the THF in proximity of the translocating polypeptide chain, but not strongly interacting with it (Fig. 5b). Biochemical data implicate residue Y743 at the fingertip in substrate movement^15,19^, and in our structure this residue is still close to the polypeptide. The single-molecule FRET data indicate that the clamp is closed while the THF retracts, and this again is consistent with our structure, in which the clamp embraces the polypeptide (Fig. 4). The closure of the clamp during ATP hydrolysis would prevent the retracting THF from dragging the polypeptide substrate backward.

The single-molecule FRET experiments also indicate that P_i_-release is the trigger for clamp opening; in the resulting ADP-bound state, the THF is disengaged and polypeptide chain can then slide in either direction^19^. The structural basis for clamp opening is likely the rotation of NBD2 outward and toward SecY^13^, which causes the two NBDs to move apart. This conformational change can be visualized by comparing the positions of NBD1 and NBD2 in crystal structures of *B. subtilis* SecA obtained in ADP·BeFx and ADP (Supplementary Fig. 7a). The same conformational change occurs in *E. coli* SecA, but is more pronounced^45^ (Supplementary Fig. 7b). The separation of the NBDs causes the release of the intercalated PPXD loop so that the preceding segment can no longer interact with the substrate^19^ (Supplementary Fig. 7c).

The current structure complements published single-molecule FRET experiments^20^, leading to a coherent model for the coordination of ATP hydrolysis with THF and clamp movements. However, our structure provides only a static snapshot of a single state, and further tests of the translocation models therefore require additional structures of the active translocation complex with different nucleotides and substrates.

## Methods

### Protein expression and purification

The expression plasmids, pTet-*G. thermodenitrificans* SecE_His8_/Y and pBAD-proOmpA-sfGFP_3Cstrep_/*B. subtilis* SecA778-Enhancer, were co-transformed into *E. coli* strain EP51^33^ (Supplementary Fig. 1). All residue numberings are for *B. subtilis* SecA and *G. thermodenitrificans* SecY and SecE. SecY contained a mutation at residue 60 (G60C). Residues 202–213 in the loop between TMs 5 and 6 of SecY were replaced by the sequence TFGGLN, as in the construct used for crystallization, a mutation that did not affect SecY activity^23^. A cysteine was introduced at the 7th residue following the signal sequence of the substrate. The expression of the SecYE was induced by the addition of 200 ng/mL anhydrotetracycline at OD600 0.8–0.9. The cells were incubated in a shaker for 1.0 h at 37 °C and 0.5 h at 22 °C. Then, 0.15% L-arabinose was added to the culture to induce the expression of substrate and SecA at 16 °C overnight. The cells were harvested and stored at −80 °C until use.

The cells were suspended in buffer A (20 mM Tris-HCl pH 7.5, 150 mM NaCl) and lysed with an Emulsiflex C3 (Avestin) homogenizer. The membranes were pelleted by ultracentrifugation, washed once with buffer B (20 mM Tris-HCl pH 7.5, 300 mM NaCl), and solubilized in 0.5% n-dodecyl β-D-maltoside (DDM; Anatrace) and 0.5% lauryl maltose neopentyl glycol (LMNG; Anatrace) in buffer C (20 mM Tris-HCl pH 7.5, 150 mM NaCl, 10% glycerol). After 1 h incubation at 4 °C, the solution was clarified by ultracentrifugation. The supernatant was mixed with 5 ml Ni-NTA resin (Thermo Fisher) and incubated for 1 h at 4 °C. After washing with 15 ml of buffer D (as buffer C, but with 0.02% LMNG) containing 10 mM imidazole and 5 ml of buffer D containing 15 mM imidazole, the protein was eluted with 5.5 ml of buffer D containing 250 mM imidazole. The eluted material was immediately loaded onto a column pre-packed with 0.75 ml StrepTactin resin (IBA). The protein was eluted with buffer C containing 0.1% digitonin (Biosynth) and 10 mM desthiobiotin. About 1.5 ml of the eluent was collected and immediately supplemented with 5 mM MgCl_2_ and 1 mM ADP·BeFx. The protein was concentrated with an Amicon filter (100 kD MWCO, EMD Millipore) and loaded onto a Superdex 200 10/300 column (GE Healthcare) in buffer G (20 mM HEPES-NaOH pH 7.0, 100 mM NaCl, 0.1% digitonin, 5 mM MgCl_2_, 1 mM ADP·BeFx). The peak fractions were concentrated to ~6 mg/ml, aliquoted, and flash-frozen in liquid nitrogen. The protein was stored at −80 °C.

The plasmid encoding an anti-SecY Nanobody with an N-terminal His-SUMO tag was transformed into *E. coli* strain BL21 (DE3). The cells were grown in LB medium at 37 °C. Protein expression was induced with 0.5 mM IPTG at OD_600_ = 0.6. The expression was continued at 20 °C overnight. About 7 g of cells were obtained from 1 L of culture. The cells were lysed by sonication. After centrifugation, the supernatant was mixed with 4 ml of Ni-NTA resin (Thermo Fisher) and incubated at 4 °C for 1 h. The protein was eluted with 250 mM imidazole, and then diluted and concentrated in buffer A with 1 mM dithiothreitol (DTT) to remove imidazole. The SUMO tag was cut off by incubating with the SUMO protease at 4 °C overnight. The sample was then applied to a Ni-NTA resin again to remove the SUMO tag. The nanobody was further purified by gel filtration on a HiLoad 16/600 Superdex 75 pg column in buffer A. The purified nanobody was concentrated to 30 mg/ml.

The MSP1D1 protein was purified according to the published protocols^46^. Briefly, the His-tagged MSP1D1 was expressed in *E. coli* strain BL21 (DE3). The cells from 1 L culture were lysed by sonication in the lysis buffer (40 mM Tris pH 8.0, 300 mM NaCl, 10% glycerol, 1% Triton-X100). The protein was purified on a Ni-NTA resin, followed by gel filtration. Finally, MSP1D1 was concentrated to 11 mg/ml in gel filtration buffer (20 mM Tris pH 7.4, 150 mM NaCl).

### Improving nanobody affinity by yeast display

GeneMorph II Random Mutagenesis Kit (Agilent) was used to introduce random mutations into the DNA sequence of AYC08, the nanobody used for determining the previous crystal structure^23^. The mutation rate was estimated to be ~6 bp/1 kb. The pool of mutated DNA sequences was co-electroporated with a linearized yeast display vector, pYDS649HM^47^, into *Saccharomyces cerevisiae* (strain: BJ5465). The display of the nanobody mutants on the yeast cell surface was induced by galactose. It was estimated that the sequence diversity of the mutant library was about 10^8^. The induced cells were stained with an anti-HA antibody labeled with Alexa647 (BioLegend) and SecA-OAIns-sfGFP/SecYE^23^. The stained cells were sorted and analyzed by flow cytometry. Nanobodies with the highest SecY affinity were enriched after several rounds of sorting. Several mutations were identified and sequenced.

### Reconstitution of the translocation complex into nanodiscs

The purified translocation complex was mixed with MSP1D1 and *E. coli* polar lipids (Avanti Lipids, 40 mg/ml dissolved in 0.5% DDM) at a molar ratio of 1:2:25. Bio-beads SM2 (Bio-Rad) were then added to the mixture and incubated at 4 °C overnight to remove the detergents. The complex was further purified on a StrepTactin resin to remove empty nanodiscs. The reconstituted and purified nanodiscs had a concentration of 1.6 mg/ml in the elution buffer (20 mM HEPES pH 7.0, 150 mM NaCl, 10 mM desthiobiotin, 5 mM MgCl_2_, 1 mM BeFx, and 0.5 mM ADP.

### Cryo-EM sample preparation and data collection

The freshly prepared nanodisc samples were mixed with the anti-SecY nanobody at a molar ratio of 1:1.2 before vitrification. Holey-carbon gold grids (Quantifoil, R1.2/1.3) were glow-discharged with a plasma cleaner, and for each grid 5 µl sample was used. Cryo-grid preparation was performed with an FEI Vitrobot Mark IV with the inner chamber set at 4 °C and 100% humidity. The cryo-grids were screened with a 200 kV FEI Talos Arctica microscope (FEI Ceta camera). Data sets were collected on a 300 kV FEI Titan Krios TEM (Gatan K2 summit camera) with GIF Quantum energy filter (Gatan). The images were collected at a dose rate of 4.8 e^−^/s/Å^2^ with an exposure time of 12 s. Movie stacks (40 frames each) were recorded with the software serial EM^48^ under low-dose conditions. The magnification was set at ×130,000 and the defocus ranged from −1.5 to −2.5 μm. Statistics for data collection was summarized in Supplementary Table 1.

### Image processing

A total of 5968 movie stacks were collected. Motion correction and electron-dose weighting were performed by using MotionCor2^49^. The program Gctf^50^ was used to estimate the contrast transfer function (CTF) parameters. Images of high quality were selected for further image processing on the basis of the CTF power spectra of the corrected images. A small set of 2093 particles were hand-picked and subjected to 2D classification using RELION3.0^51^. Class average images of high quality were selected and used as templates for particle auto-picking with RELION3.0. After two rounds of 2D classification (Supplementary Fig. 2), 472,724 particles (two batches of data) were selected for 3D classification. The initial three-dimensional (3D) model was calculated using cisTEM^52^. For the first batch of 290,961 particles, a cascade of 3D classification with a binning factor of two was applied to further exclude bad particles (Supplementary Fig. 2c). Among the eight groups from the first round of 3D classification, two with good secondary structure features were combined (108,684 particles) for a second round of 3D classification. Based on the map appearance and the resolution of secondary features, three classes (56,857 particles) were kept for 3D refinement. The second batch of data (181,763 particles) was similarly processed. A final set of 130,153 particles from both data batches were combined for refinement that resulted in maps of resolutions of 3.88 and 3.50 Å after mask-based post-processing. The particles were subjected to further CTF and 3D refinement. The final resolution is 3.82 Å without post-processing and 3.45 Å after mask-based post-processing (Supplementary Figs 2d and e). All the resolution estimations were based on gold-standard Fourier Shell Correlation (FSC) 0.143 criteria. We also tried to classify the particles using mask-based classification focusing on regions of the peptide substrate. However, only marginal improvement was achieved.

Models for SecA-OAIns/SecYE/AYC08 (PDB ID 5EUL) and GFP-enhancer (PDB ID 3K1K) were fit into the electron density map. The polypeptide substrate, the plug, SecE, the helical wing domain of SecA, and some regions at the SecA-SecY interfaces were rebuilt. The model was refined in real space using Phenix^53^. Model validation was done with MolProbity^54^.

## Supplementary information

Supplementary Information

Reporting summary

## Data Availability

The cryo-EM maps have been deposited in the Electron Microscopy Data Bank under accession numbers EMD-9731. The atomic structure coordinates have been deposited in the Protein Data Bank under the accession number 6ITC. All other data can be obtained from the corresponding author upon reasonable request.
