# Supplementary material for: Structure of the substrate-engaged SecA-SecY protein translocation machine
Source: Nat Commun. 2019 Jun 28;10:2872. doi: 10.1038/s41467-019-10918-2 (PMC6599042; doi:10.1038/s41467-019-10918-2)
Supplement: Supplementary file 1 — Supplementary Information [file 41467_2019_10918_MOESM1_ESM.pdf]

Supplementary Information

Structure of the substrate-engaged SecA-SecY protein translocation machine

Ma *et al*

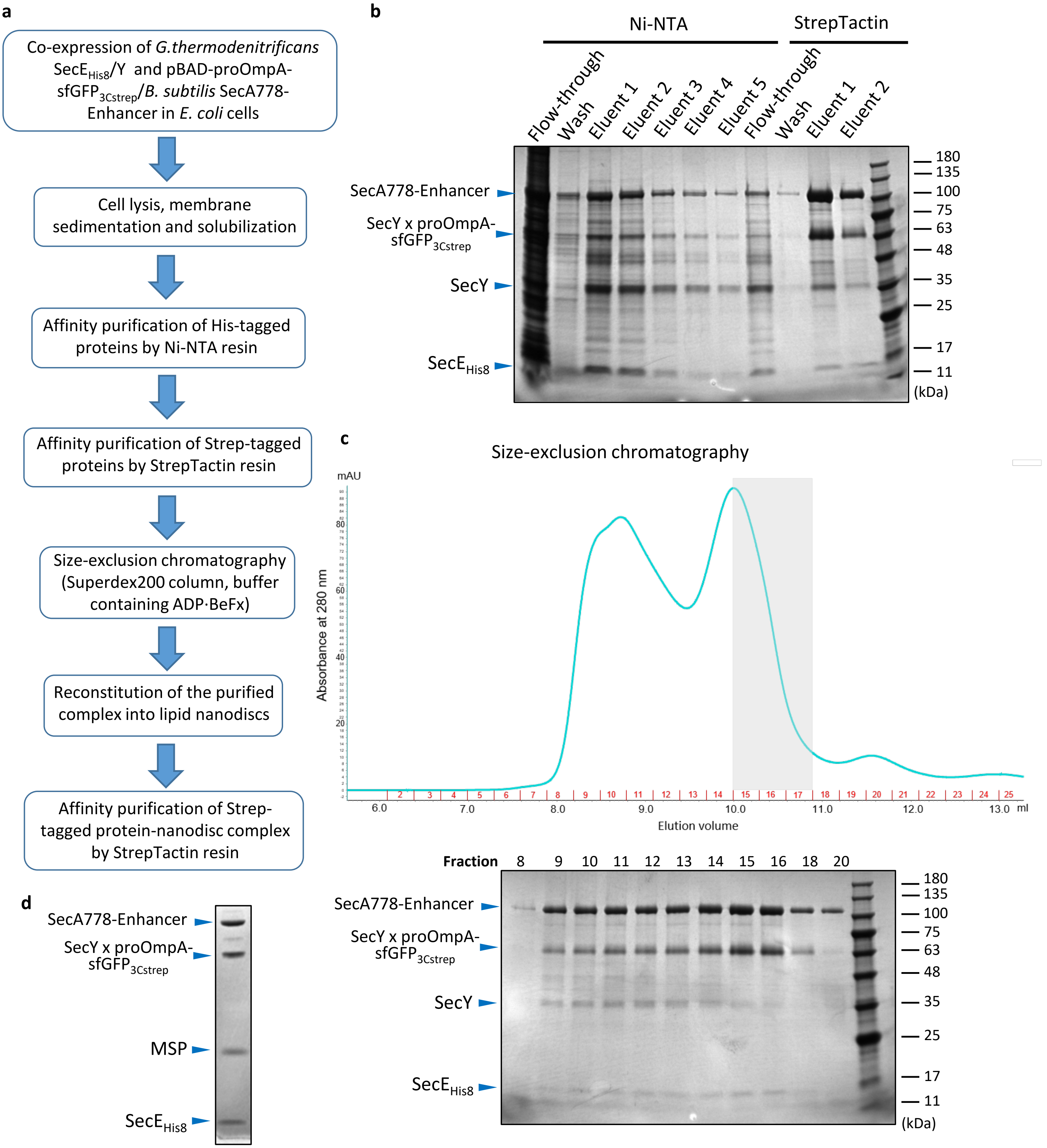

Supplementary Figure 1. Purification of an active translocation complex.
a. Flow-chart of protein expression and purification. **b.** SDS-PAGE analysis of samples taken during affinity purification with Ni-NTA and StrepTactin resins. **c.** Elution profile and SDS-PAGE analysis of fractions obtained during protein purification with a Superdex200 column. The shaded area indicates fractions that were combined for subsequent nanodisc reconstitution. **d.** SDS-PAGE of the purified protein translocation complex reconstituted into nanodiscs, which was used for cryo-EM analysis.

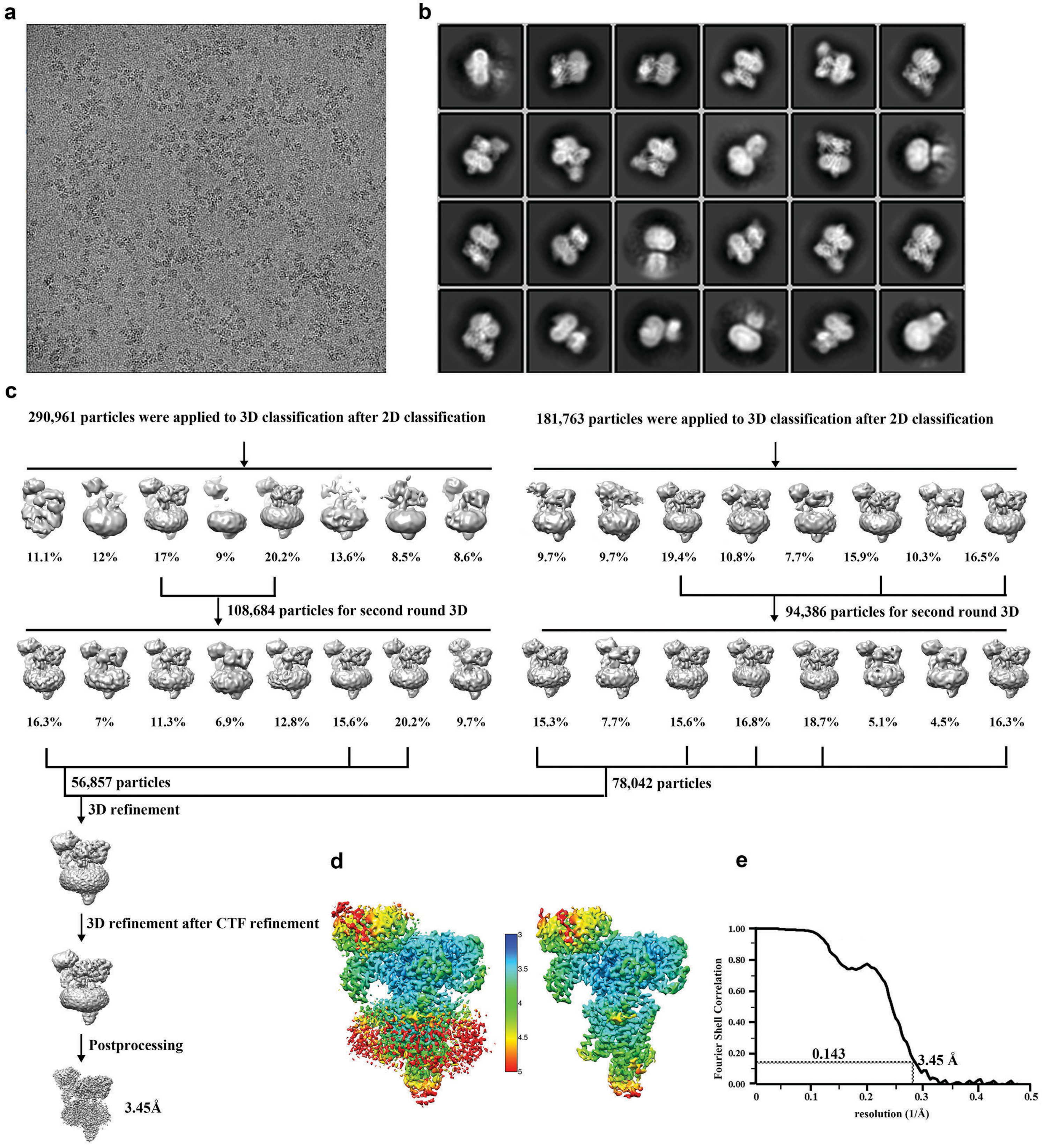

Supplementary Figure 2. Cryo-EM structure determination of the SecA-SecY- substrate complex.
a. Shown is a representative cryo-EM image collected with a Titan Krios 300 kV microscope. **b.** Selected 2D classes of the particles. **c.** 3D classification and refinement workflow. **d.** Local resolution map of the final, sharpened density map shown with (left) or without (right) the nanodisc. **e.** Fourier shell correlation (FSC) curve with estimated resolution according to the gold standard.

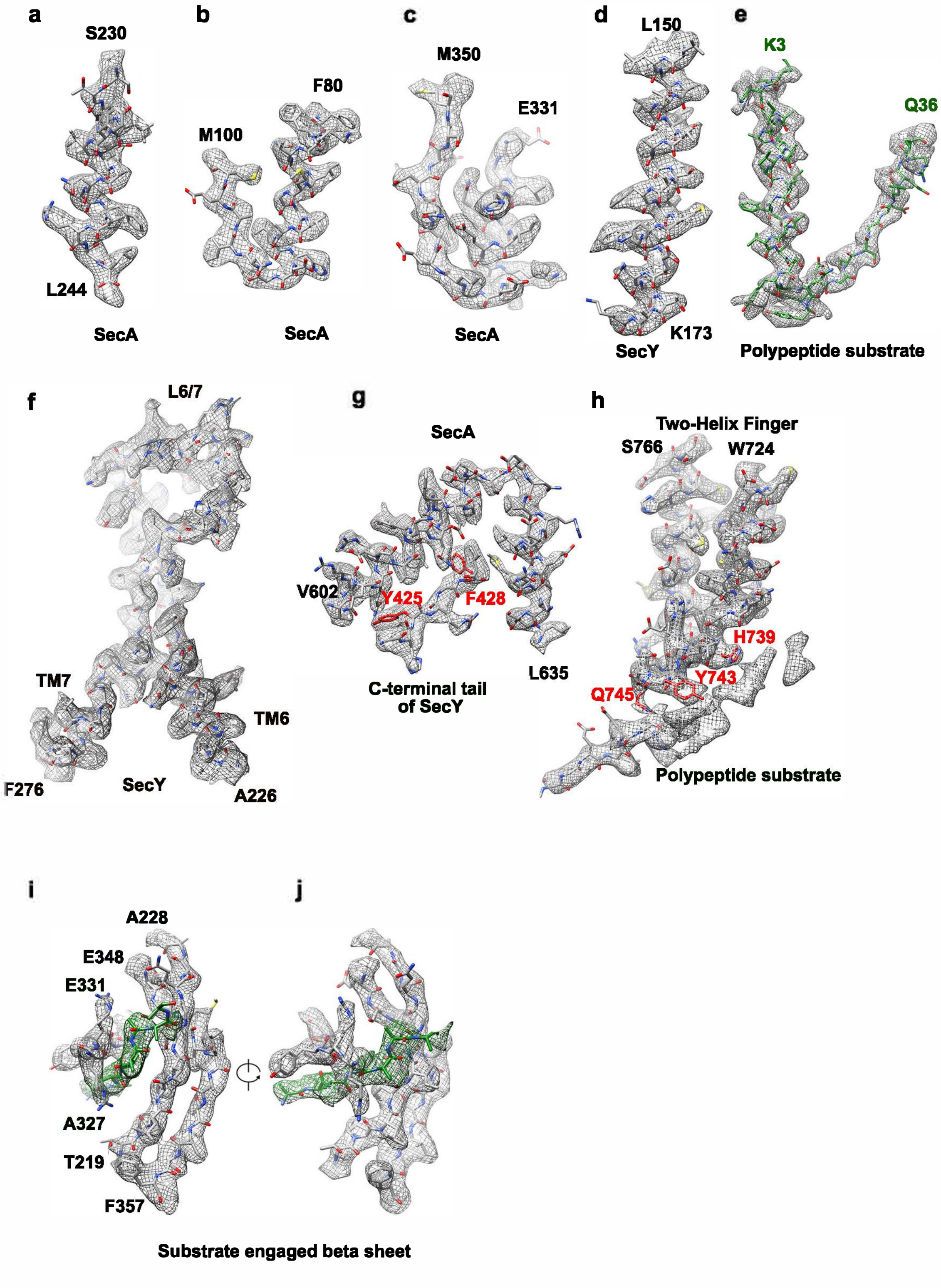

Supplementary Figure 3. Examples of the fit of models into the density map.
a-e. Density map and model in selected regions of SecA, SecY, and the polypeptide substrate including the signal sequence. Residues at the beginning and end of each polypeptide segment are indicated. **f.** Density map of the loop between TMs 6 and 7 of SecY (L6/7). **g.** Density map of the C-terminal tail of SecY. Two aromatic residues in the tail are colored in red. **h.** Density map of the THF region. Three well-resolved THF residues involved in substrate interaction are colored in red. **i** and **j.** Electron density maps of the β-sheet region. The substrate is colored in green.

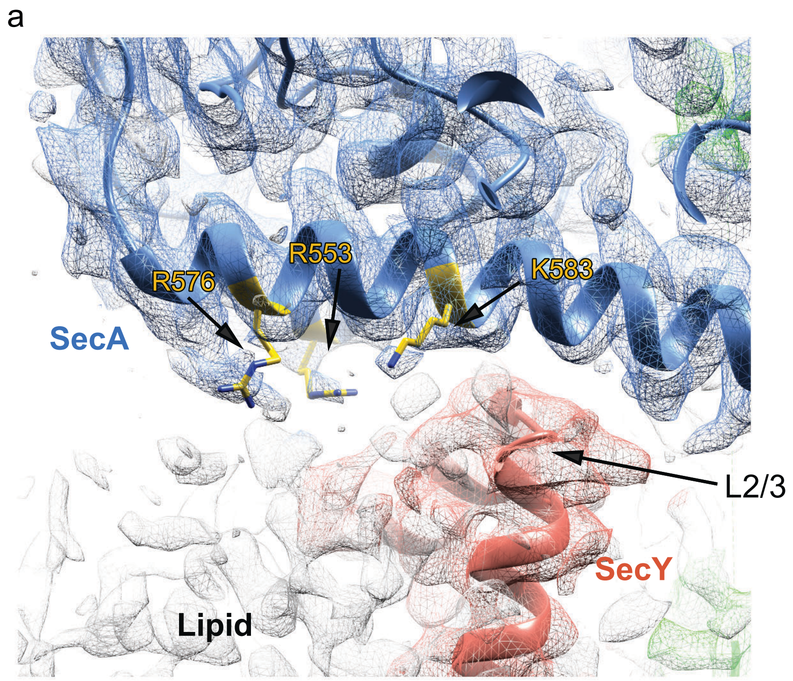

Supplementary Figure 4. Interaction of SecA with the lipid bilayer surface.
a. SecA residues involved in lipid interactions close to the N-terminal half of SecY are shown as sticks and colored yellow. The loop between TMs 2 and 3 of SecY (L2/3) is labeled. Density is shown as a mesh and colored red, blue, and grey for SecY, SecA, and lipids, respectively. **b.** As in **a**, but shown are SecA residues involved in the lipid interactions close to the C-terminal half of SecY.

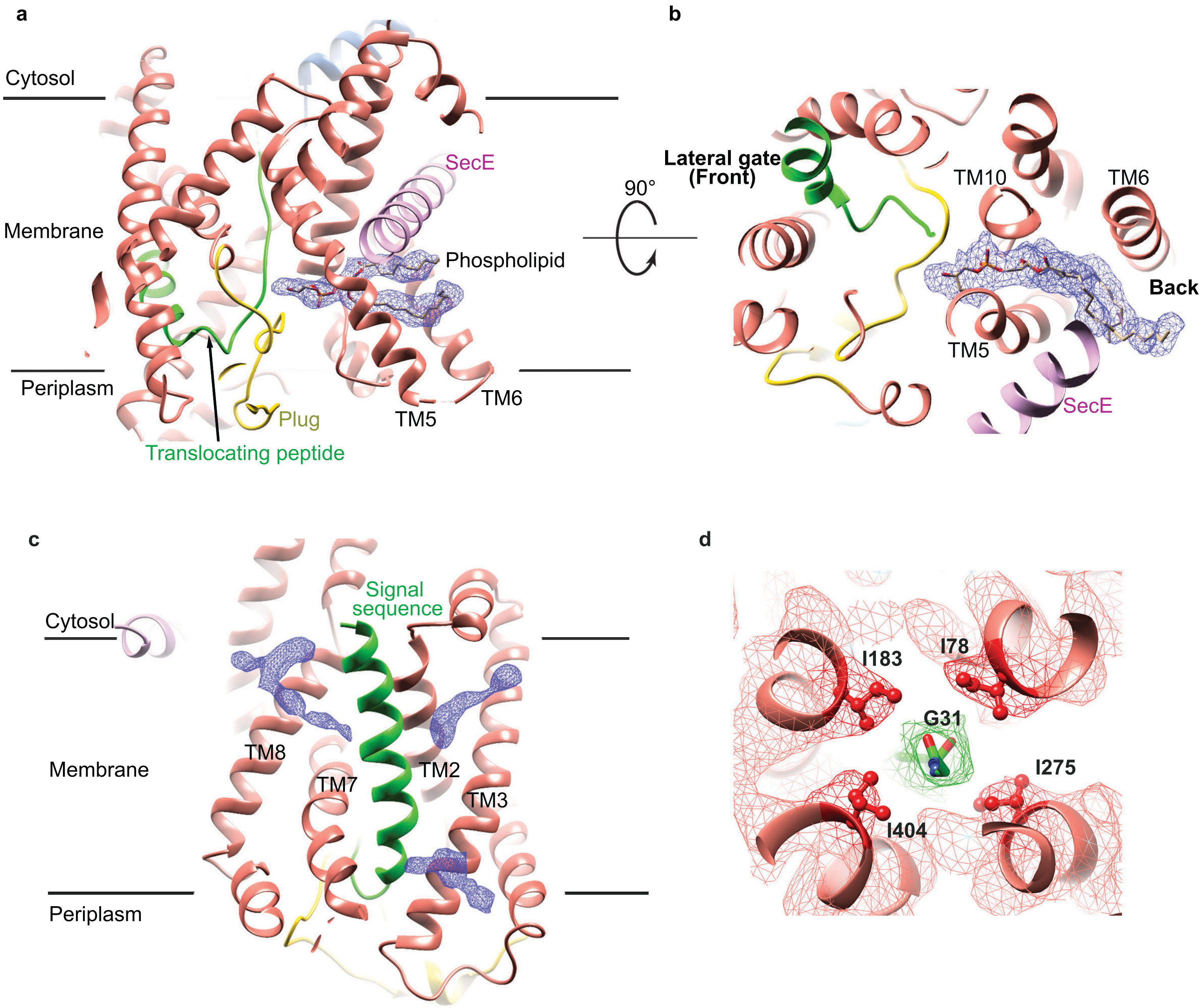

Supplementary Figure 5. Phospholipid molecules and the substrate in the SecY channel.
a. Side view of SecY with the density of a central phospholipid molecule shown as a blue mesh and the corresponding model shown as sticks. **b.** As in **a**, but top view. **c.** Electron density blobs (blue meshes) that probably correspond to lipid molecules. The lipids interact with both the signal sequence (green) and SecY. **d.** Top view of the pore ring residues (red) forming a seal around the translocating polypeptide (green), with Gly31 of the substrate trapped in the ring. Pore ring residues are shown as balls and sticks and density as meshes.

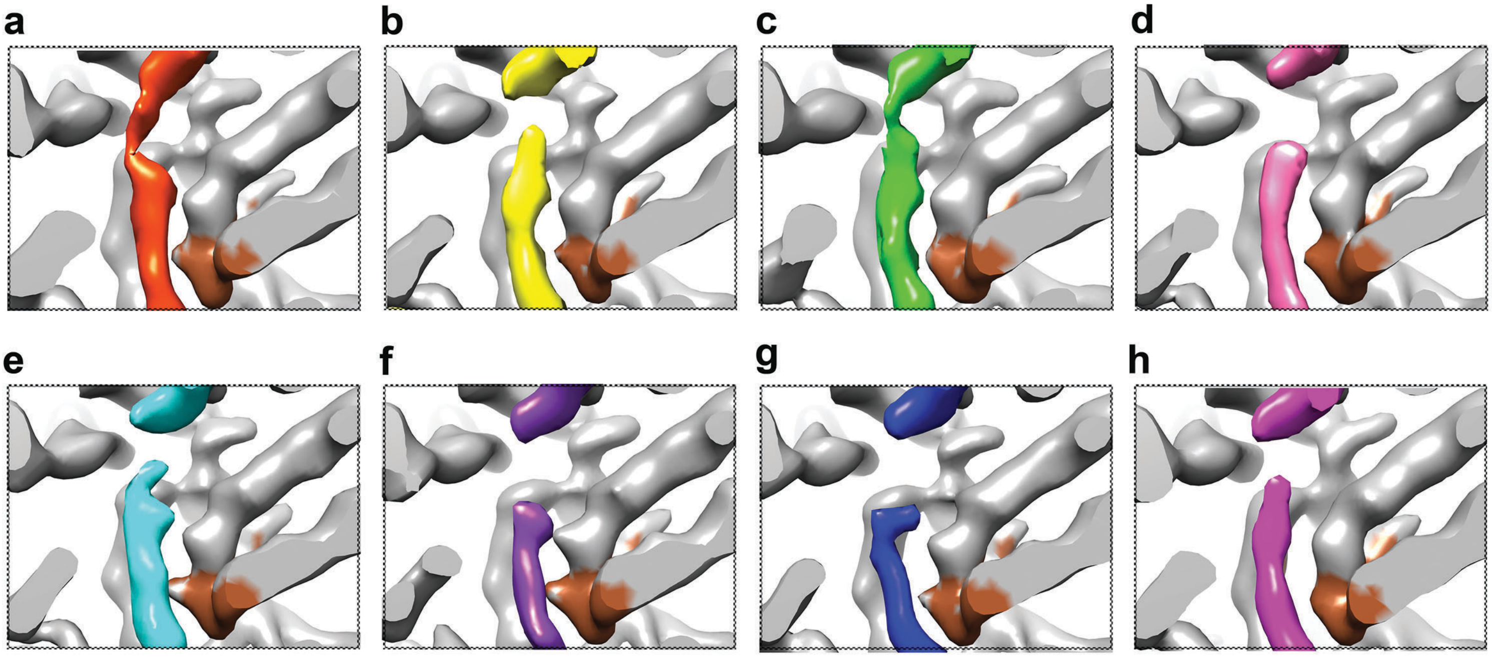

Supplementary Figure 6. Multiple conformations of the substrate close to the THF.
a-h. Shown is the THF region in eight unsharpened density maps from 3D classification. Density for the tip of the THF is colored brown. The density of the polypeptide substrate is shown in different colors. The maps are displayed at similar contour levels.

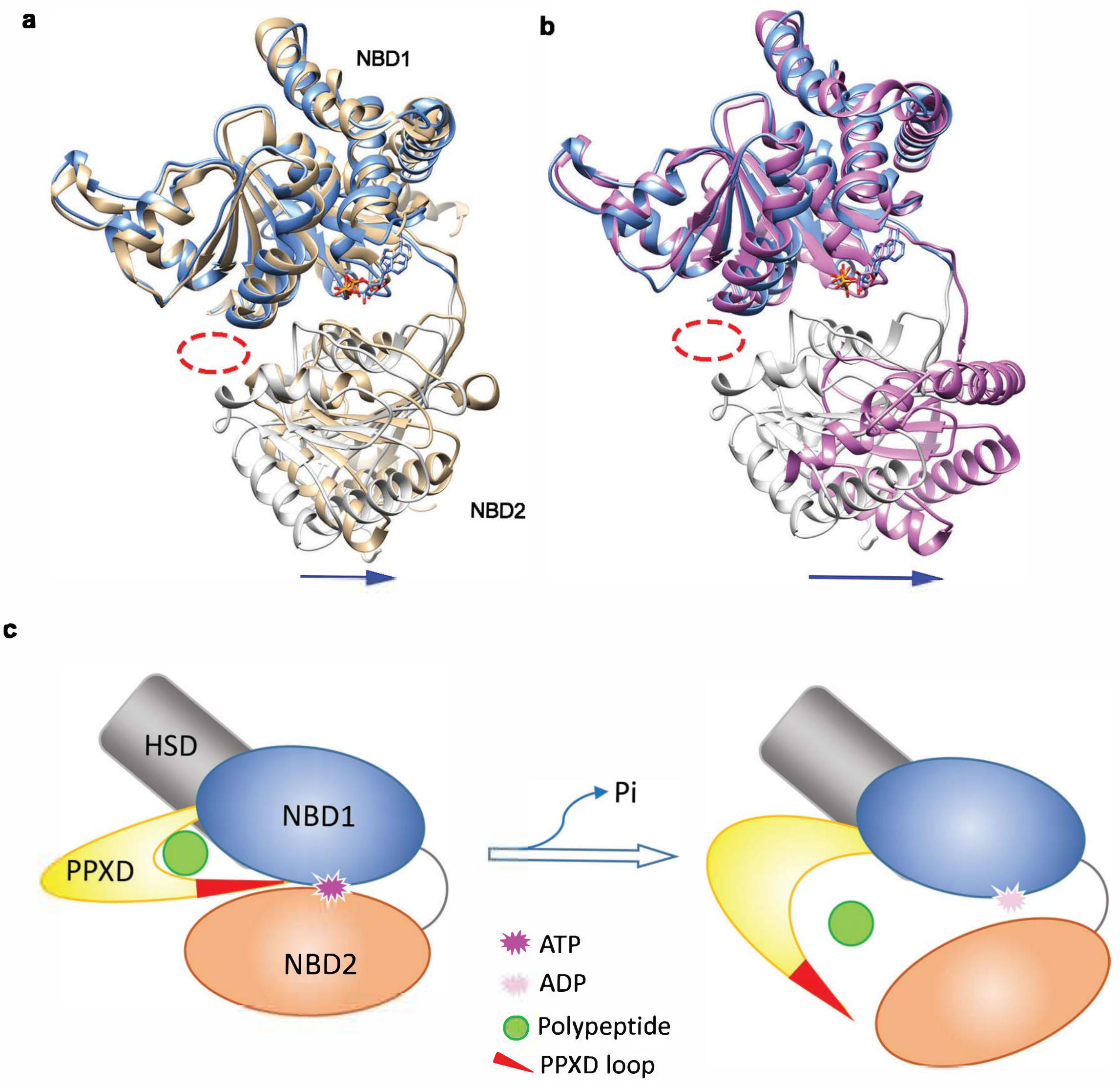

Supplementary Figure 7. Relative movements of SecA's NBDs during ATP hydrolysis.
a. Opening of the clamp in the ADP-bound state of SecA. Shown are the NBD1 and NBD2 domains in the cryo-EM structure (ADP.BeFx state; blue and light grey, respectively) and in a structure of *B.subtilis* SecA in the ADP state (PDB ID: 1M74; both NBDs in tan). The alignment was done on the basis of NBD1 and the movement of NBD2 after ATP hydrolysis is indicated by an arrow. The red dashed oval indicates the cavity formed between NBD1 and NBD2, which is occupied by the PPXD loop in the cryo-EM structure and is open in the ADP state. ADP-BeFx and ADP are shown as sticks. **b.** As in **a**, but comparison with *E.coli* SecA in the ADP state (PDB ID: 2FSI, both NBDs in purple). **c.** Model for the mechanism of clamp opening. Pi-release separates NBD1 and NBD2, which releases the intercalated PPXD loop, resulting in the clamp no longer holding the substrate.

Supplementary Table 1. Cryo-EM data collection, refinement, and validation statistics

	SecA-SecYE-proOmpA-sfGFP (EMDB-9731, PDB 6ITC)
**Data collection and processing**	
Magnification	130,000
Voltage (kV)	300
Electron exposure (e–/Å^2)	57.6
Defocus range (μm)	-1.5 to -2.5
Pixel size (Å)	1.05
Symmetry imposed	C1
Initial particle images (no.)	472,724
Final particle images (no.)	130,153
Map resolution (Å)	3.45
FSC threshold 0.143	0.143
Map resolution range (Å)	3.2-5.0
**Refinement**	
Initial model used (PDB code)	5EUL, 3K1K
Model resolution (Å)	3.45
FSC threshold	0.143
Model resolution range (Å)	3.45
Map sharpening *B* factor (Å^2)	-120
Model composition	
Non-hydrogen atoms	13856
Protein residues	13729
Ligands	127
B factors (Å^2)	
Protein	72.6
Ligand	74.4
R.m.s. deviations	
Bond lengths (Å)	0.012
Bond angles (°)	1.379
Validation	
MolProbity score	1.94
Clashscore	7.48
Poor rotamers (%)	0.68
Ramachandran plot	
Favored (%)	90.79
Allowed (%)	8.86
Disallowed (%)	0.35
